# Two New C21 Steroidal Glycosides from the Roots of *Cynanchum paniculatum*

**DOI:** 10.1007/s13659-019-0205-2

**Published:** 2019-04-26

**Authors:** Hai-Li Yu, Qin Long, Wen-Fang Yi, Bao-Jia Yang, Yu Song, Xiao Ding, Shun-Lin Li, Xiao-Jiang Hao

**Affiliations:** 1 State Key Laboratory of Phytochemistry and Plant Resources in West China, Kunming Institute of Botany, Chinese Academy of Sciences, Kunming, 650201 People’s Republic of China; 2 University of Chinese Academy of Sciences, Beijing, 100049 People’s Republic of China

**Keywords:** *Cynanchum paniculatum*, Steroidal glycosides, Bioactivities, NMR data

## Abstract

**Abstract:**

Two new C21 steroidal glycosides, paniculatumosides H and I, together with four known ones were isolated from the roots of *Cynanchum paniculatum* (Bge.) Kitag. Their structures were identified by spectroscopic methods including extensive 1D and 2D NMR techniques. All compounds were subjected to detect the anti-tobacco mosaic virus (TMV) activities and their cytotoxities against three human tumor cell lines (SMMC-7721, MDA-MB-231 and A549). The results showed that compounds **1** and **5** exhibited potent protective activities against TMV, while **2**, **4** and **6** had moderate effects on the SMMC-7721 cancer cells viability.

**Graphical Abstract:**

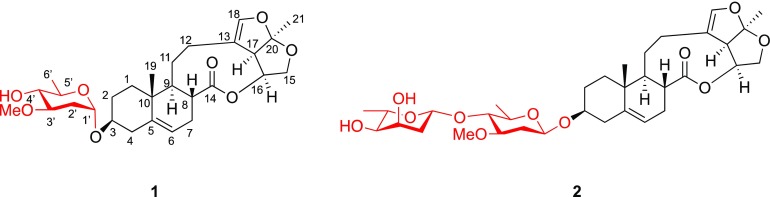

## Introduction

**Fig. 1:**
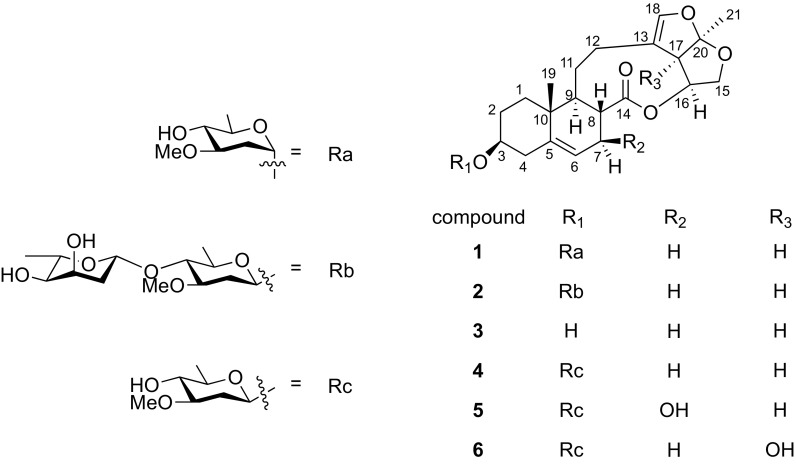
Chemical structures of compounds **1**–**6**

*Cynanchum paniculatum* (Bunge) Kitag is a vivacious herb broadly distributed in China, Japan and Korea, whose dried roots have been used as a Chinese herbal medicine for the treatment of rheumatic arthralgia, epigastric pain, toothache, lumbago, traumatic injuries, and eczema [1–3]. In the previous work, it has been confirmed that *C. paniculatum* contains C_21_ steroidal glycosides [4, 5]. Pregnanes and their glycosides have shown many aspect activities, such as antitumor, antifungal, antiviral and cytotoxic activities [6, 7]. Our previous works found that pregnanes and their glycosides could inhibit tobacco mosaic virus through suppressing the expression of viral subgenomic RNA but without affecting the accumulation of viral genomic RNA [6]. Therefore, in order to find the structurally unique natural products in *C. paniculatum* and explore their biological activities, we investigated the dichloromethane extract of *C. paniculatum*, and two new steroidal glycosides paniculatumosides H (**1**) and I (**2**), together with four known ones glaucogenin C (**3**) [8], cynatratoside A (**4**) [8], cynapanoside A (**5**) [9] and neocynapanogenin F 3-O-*β*-d-oleandropyranoside (**6**) [10] were isolated. Furthermore, we tested the anti-TMV activities and cytotoxicity of these compounds (Fig. 1).

## Result and Discussion

### Structure Elucidation

**Fig. 2:**
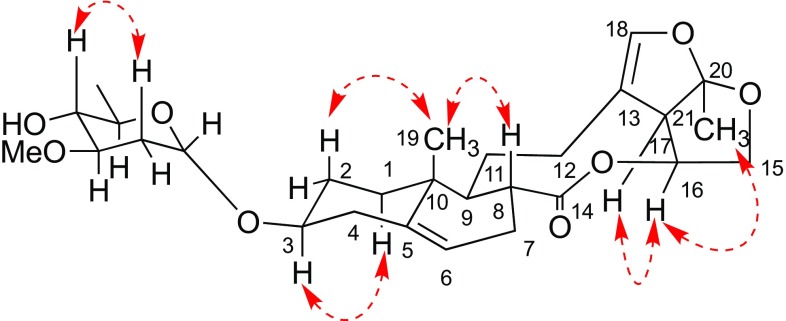
The key ROESY correlations of **1**

**Fig. 3:**
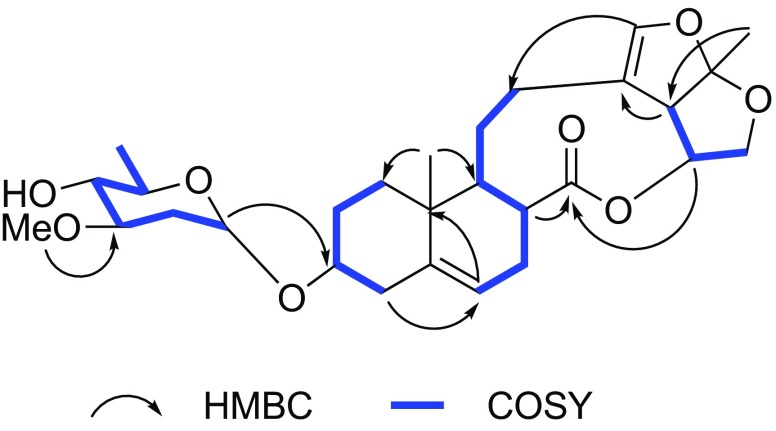
The selected HMBC and COSY correlations of **1**

Paniculatumoside H (**1**) was obtained as a yellow powder. Its molecular formula, C_28_H_40_O_8_, was determined by analysis the peak at *m/z* 527.2629 [M + Na]^+^ in the positive HRESI-MS (calcd for C_28_H_40_O_8_Na 527.2615). The IR spectrum displayed absorption bands for hydroxyl (3443 cm^−1^), carbonyl (1736 cm^−1^), olefinic (1635 cm^−1^) and carbon–oxygen bond (1081 cm^−1^). The ^1^H NMR spectrum of compound **1** showed the presence of two tertiary methyl groups at *δ*_H_ 0.92 (3H, s, H-19) and *δ*_H_ 1.53 (3H, s, H-21), one olefinic proton at *δ*_H_ 5.38 (d, *J* = 5.3 Hz, H-6), one olefinic deshielded proton at *δ*_H_ 6.24 (s, H-18), two oxygen-substituted methine protons at *δ*_H_ 3.44 (m, H-3) and *δ*_H_ 5.29 (td, 9.5, 7.1, H-16) and two methylene protons at *δ*_H_ 3.84 (t, *J* = 8.6 Hz, H-15a) and *δ*_H_ 4.15 (td, *J* = 8.6, 1.4 Hz, H-15b). By comparison of its ^1^H and ^13^C NMR spectra (Table 1) to those of glaucogenin C (**3**) indicated that the aglycone of **1** is glaucogenin C, which also confirmed by correlations of H-19/H-2b, H-19/H-8, H-1b/H-3, H-21/H-16 and H-16/H-17 in the ROESY experiment (Fig. 2). The main difference was an extra sugar unit present at **1**, which could be further verified by the glycosidation shifts at C-2 (− 3.8), C-3 (+ 4.2) and C-4 (− 2.2). The sugar carbon signals (*δ*_C_ 95.2, 78.2, 76.8, 67.5, 56.5, 34.5, 17.8) illustrated that the sugar belonged to 2,6-deoxy sugar because it contained four methines, one methylene, a terminal methyl group and a methoxyl group. In the sugar spin systems, the large coupling constants *J*_H-3′/H-4′_ (9.1 Hz) and *J*_H-4′/H-5′_ (9.3 Hz) disclosed their *trans*-diaxial relationship, which suggested that the sugar was oleandropyranose [8]. Moreover, the anomeric proton of d-oleandrose was *α*-orientation based on the small coupling constants of H-1′ (^3^*J*_H-1,H-2_ = 3.5 Hz) [11]. Furthermore, the sugar carbon and proton signals were identical with *α*-d-oleandropyranosyl unit by comparison with reported literature [12], and the ROESY correlations of H-2′b/H-4′ supported this configuration (Fig. 2). Thus, compound **1** could be glycosylated at the C-3 with *α*-linkage by d-oleandropyranosyl unit, which was also concluded from the HMBC correlation of *δ*_H_ 5.04 (d, *J* = 3.5 Hz, H-1′) to *δ*_C_ 75.8 (C-3) (Fig. 3). The structure of **1** was finally established as glaucogenin C 3-*O*-*α*-d-oleandropyranoside.

**Table 1:** ^1^H NMR (500 MHz) and ^13^C NMR (125 MHz) spectroscopic data for compounds **1**, **2** and **3** in CDCl_3_

No.	**1**		**2**		**3**	
	*δ*_C_	*δ*_H_	*δ*_C_	*δ*_H_	*δ*_C_	*δ*_H_
1a 1b	36.2	1.96 (m) 1.02 (td, 14.4, 3.8)	36.4	1.96 (m) 1.05 (td, 14.6, 3.9)	36.4	1.96 (m) 1.07 (td, 13.7, 3.3)
2a 2b	27.5	1.91 (m) 1.45 (m)	29.4	1.97 (m) 1.60 (m)	31.3	1.86 (m) 1.51 (m)
3	75.8	3.44 (m)	77.9	3.53 (m)	71.6	3.53 (m)
4a 4b	39.7	2.30 (m) 2.24 (m)	38.6	2.33 (m) 2.16 (m)	41.9	2.32 (dd, 13.0, 2.5), 2.17 (dd, 13.0, 11.9)
5	140.3	–	140.2	–	140.2	–
6	120.2	5.38 (d, 5.3)	120.3	5.39 (d, 5.2)	120.2	5.39 (d, 5.3)
7a 7b	28.0	2.43 (m) 2.05 (m)	28.0	2.42 (m) 2.04 (m)	27.9	2.43 (m) 2.02 (m)
8	40.4	2.42 (m)	40.4	2.42 (m)	40.4	2.43 (m)
9	52.8	1.19 (overlapped)	52.9	1.19 (overlapped)	52.8	1.20 (t, 9.7)
10	38.5	–	38.6	–	38.3	–
11a 11b	23.6	2.56 (m) 1.29 (m)	23.6	2.56 (m) 1.28 (m)	23.6	2.57 (m) 1.27 (m)
12a 12b	29.6	2.06 (m) 1.33 (m)	29.6	2.07 (m) 1.33 (m)	29.6	2.07 (m) 1.33 (m)
13	118.1	–	118.1	–	118.1	–
14	175.6	–	175.6	–	175.6	–
15a 15b	67.6	4.15 (td, 8.6, 1.4) 3.84 (t, 8.6)	67.6	4.15 (td, 7.8, 1.6), 3.84 (t, 7.8)	67.5	4.16 (td, 8.1, 1.6), 3.85 (t, 8.1)
16	75.1	5.29 (td, 9.5,7.1)	75.1	5.29 (td, 9.7, 7.3)	75.0	5.29 (td, 9.7, 7.8)
17	55.7	3.43 (overlapped)	55.7	3.43 (overlapped)	55.7	3.43 (d, 7.8)
18	143.3	6.24 (s)	143.3	6.25 (s)	143.3	6.25 (s)
19	18.0	0.92 (s)	18.0	0.91 (s)	18.0	0.91 (s)
20	114.0	–	113.9	–	113.9	–
21	24.6	1.53 (s)	24.6	1.53 (s)	24.5	1.53 (s)
Sugar						
1′	95.2	5.04 (d, 3.5)	97.8	4.53 (d, 9.6)		
2′a 2′b	34.5	2.22 (m) 1.50 (m)	36.5	2.26 (m) 1.53 (m)		
3′a	78.2	3.52 (ddd, 9.1, 8.8, 4.9)	79.1	3.38 (m)		
4′	76.8	3.15 (dd, 9.3, 9.1)	82.5	3.19 (dd, 9.0, 8.8)		
5′	67.5	3.73 (dq, 9.3, 6.3)	71.1	3.29 (m)		
6′	17.8	1.28 (d, 6.3)	18.2	1.30 (d, 6.3)		
3′-OCH_3_	56.5	3.39 (s)	56.4	3.40 (s)		
1′′			98.5	5.02 (d, 9.7)		
2′′			38.2	2.12 (m) 1.72 (m)		
3′′			68.3	4.11 (m)		
4′′			72.9	3.31 (m)		
5′′			69.6	3.74 (dq, 9.7, 6.3)		
6′′			18.4	1.31 (d, 6.3)

**Fig. 4:**
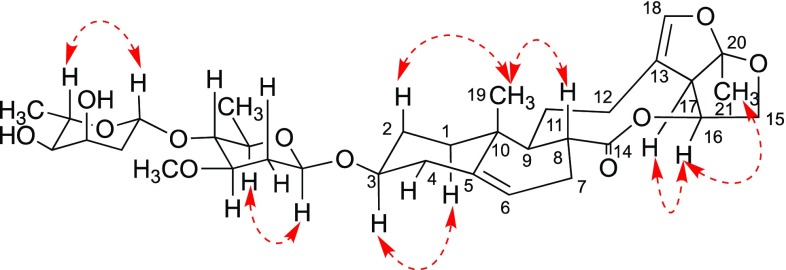
The key ROESY correlations of **2**

**Fig. 5:**
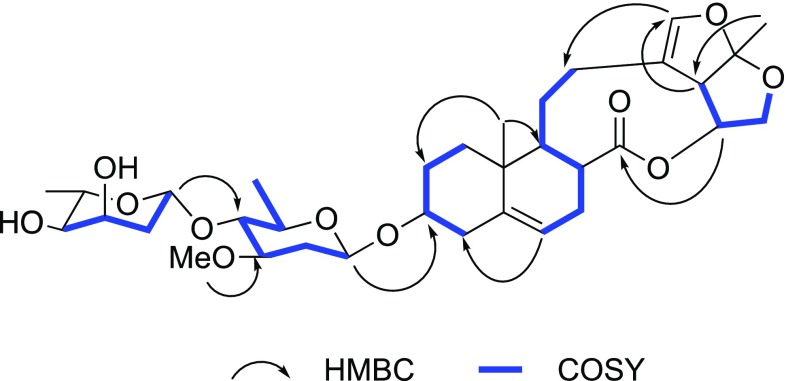
The selected HMBC and COSY correlations of **2**

Paniculatumoside I (**2**) was obtained as a colourless powder. The molecular formula of **2**, C_34_H_50_O_11_, was deduced by HRESI-MS (*m/z* 657.3258 [M + Na]^+^, calcd for C_34_H_50_O_11_Na 657.3245). The IR spectrum also suggested the presence of hydroxyl (3433 cm^−1^), carbonyl (1736 cm^−1^), olefinic (1632 cm^−1^) and carbon–oxygen bond (1070 cm^−1^). Compounds **2** and **1** possess the same aglycone by analyzing their ^1^H and ^13^C NMR spectra (Table 1), which was confirmed to be glaucogenin C by detailed analysis of their 2D NMR spectra. Two anomeric proton signals *δ*_H_ 4.53 (d, *J *= 9.6 Hz, H-1′) and *δ*_H_ 5.02 (d, *J *= 9.7 Hz, H-1′′), which correlated to the corresponding anomeric carbon signals at *δ*_C_ 97.8 (C-1′) and 98.5 (C-1′′) in the HSQC spectrum, respectively. It showed that the sugar moiety of **2** was made up of two monosaccharides. Meanwhile, from the HSQC, HSQC-TOCSY, and ^1^H, ^1^H-COSY experiments, the spin system of each monosaccharide could be established. Two methylenes (*δ*_C_ 36.5 and 38.2) and methyl groups (*δ*_H_ 1.30 and 1.31) suggested both monosaccharides of **2** were deoxysugars. The splitting patterns and the coupling constants of H-1′ (^3^*J*_H-1,H-2_ = 9.6 Hz) and H-1″ (^3^*J*_H-1,H-2_ = 9.7 Hz) illustrated that the anomeric protons of the two sugar were *β*-oriented, which was also proved by the ROESY collations between *δ*_H_ 4.53 (d, *J *= 9.6 Hz, H-1′) and *δ*_H_ 3.29 (m, H-5′), *δ*_H_ 5.02 (d, *J *= 9.7 Hz, H-1′′) and *δ*_H_ 3.74 (dq, H-5′′) (Fig. 4). The oligosaccharide moiety was identified as *β*-d-oleandropyranoside and *β*-l-digitoxopytanoside by analyzing from comparing the ^1^H and ^13^C signals of **2** with those in the literatures [6, 13]. Compound **2** was glycosylated at the C-3, which was concluded from the HMBC correlation of *δ*_H_ 4.53 (H-1′) to *δ*_C_ 77.9 (C-3), and the HMBC correlation of *δ*_H_ 5.02 (H-1′′) of *δ*_C_ 82.5 (C-4′) suggested digitoxopytanose connected to C-4′ of oleandropyranose (Fig. 5). Therefore, compound **2** was established to be glaucogenin C 3-*O*-*β*-l-digitoxopytanosyl-(1 → 4)-*β*-d-oleandropyranoside.

### Bioactivities

#### Anti-TMV Activities

All compounds were tested for anti-TMV activity using the half-leaf method. The results revealed that compounds **1** and **5** exhibited protective activities at concentration of 200 μM, with the antiviral inhibition rates of 62.7% and 59.5%, respectively. Ningnanmycin, which was used as a positive control, showed inhibition rates of 57.3%.

#### Cytotoxic Activities

In order to evaluate whether these compounds have any biological functions on cancer cells, we tested them on SMMC-7721, MDA-MB-231 and A549 human cancer cell lines for their impact on tumor cell growth by MTT method. All the compounds have no inhibitory effect on the MDA-MB-231 and A549 cell lines. But compounds **2**, **4** and **6** have moderate effects on the SMMC-7721 cancer cells viability with the IC_50_ values of 27.4 ± 0.24 μM, 22.2 ± 0.11 μM, 27.2 ± 0.13 μM, respectively. Cisplatin was used as a positive control and its IC_50_ for the SMMC-7721 cancer cells was 13.07 ± 0.27 μM.

## Experimental

### General Experimental Procedures

UV spectra were measured with a Shimadzu UV-2401A spectrophotometer. Optical rotations were determined on a Jasco P-1020 polarimeter. Infrared spectroscopy (IR) spectra were measured on a Bio-Rad FTS-135 spectrometer with KBr pellets. HRESIMS data were collected on a triple quadrupole mass spectrometer. 1D and 2D NMR spectra were recorded on a Bruker spectrometer with tetramethylsilane as the internal standard. Preparative HPLC separations were carried out using an Agilent 1200 liquid chromatograph with a Waters X-select CSH Prep RP C18 (19 × 150 mm) column and the flowing rate is 8 mL/min. Semipreparation HPLC separations were performed on an Agilent 1100 liquid chromatograph using a YMC-Pack CDS-A (10 × 250 mm) column with flowing rate of 3 mL/min. Sephadex LH-20 (40–70 mm, Amersham Pharmacia Biotech AB, Uppsala, Sweden) and Silica gel (100–200 mesh and 300–400 mesh, Qingdao Marine Chemical, Inc., Qingdao, P. R. China) were used for column chromatography.

### Plant Material

The roots of *C. paniculatum* were purchased from a medicinal market (Kunming luosiwan Chinese herbal medicine market) in August 2017 and identified by prof. Hua Peng of Kumming Institute of Botany, Chinese Acdemy of Sciences (CAS).

### Extraction and Isolation

The roots of *C. paniculatum* (100.0 kg) were powdered and extracted three times with MeOH at room temperature to afford 7.2 kg of crude extract. The extract was partitioned between CH_2_Cl_2_ and aqueous solution portions which yielded 4.4 kg crude CH_2_Cl_2_ extract. This extract was subjected to normal-phase silica gel column chromatography eluted with a gradient of petroleum ether–acetone (from 1:0 to 1:2) and CH_2_Cl_2_–MeOH (10:1–0:1) to obtain eight major fractions (Fr.1-8). Fr.6 (182.3 g) was separated by reversed-phase separation (CH_3_OH–H_2_O, 4:6–9:1) to get twelve subfractions (Fr.6a-6l). Fr.6g (2.1 g) was purified by Sephadex LH-20 eluting with MeOH to yield three fractions. Fr.6g-2 (161.4 mg) was applied to a silica gel column using CH_2_Cl_2_–MeOH (200:1–0:1) to obtain **3** (7.1 mg). Fr.6g-3 (92.5 mg) was subject to a normal-phase column chromatography and further purified by semiprepative HPLC (68% CH_3_CN in water) to yield **6** (5.0 mg, t_*R*_ = 15.0 min). Fr6I (4.3 g) was separated into four subfractions by Sephadex LH-20 eluting with CH_2_Cl_2_–MeOH (1:1). Fr.6I-2 (1.9 g) was chromatographed on a silica gel column eluting with CH_2_Cl_2_–MeOH (200:1–0:1) to get Fr.6I-2c (109.2 mg) and Fr.6I-2e (79.3 mg). Fr6I-2c was purified by semiprepative HPLC (50% CH_3_CN in water) to obtain **1** (5 mg, t_*R*_ = 27.5 min) and **4** (92.0 mg, t_*R*_ = 29.0 min). Fr.6I-2d also used semiprepative HPLC (46% CH_3_CN in water) to yield **5** (10.2 mg, t_*R*_ = 35.0 min). Fr.6 k (3.6 g) was fractioned by a silica gel column eluting with petroleum ether-acetone (80:1–0:1) to afford the Fr6 k-3 (39.3 mg), which was purified by Sephadex LH-20 (MeOH) and semiprepative HPLC (49% CH_3_CN in water) in sequence to yield **2** (8.2 mg, t_*R*_ = 31.5 min).

#### Paniculatumoside H (**1**)

White amorphous powder; [*α*]_D_^20^−21.5 (*c* 0.11, MeOH); IR (KBr) *ν*_max_ 3443 (OH), 2919, 1736, 1635, 1384, 1081 cm^−1^; ^1^H and ^13^C NMR data, see Table 1; ESIMS *m/z* 527 [M + Na]^+^; HRESIMS *m/z* 527.2629 [M + Na]^+^ (calcd for C_28_H_40_O_8_Na, 527.2615).

#### Paniculatumoside I (**2**)

White amorphous powder; [*α*]_D_^20^−52.0 (*c* 0.09, MeOH); IR (KBr) *ν*_max_ 3433 (OH), 2922, 1736, 1632, 1383, 1070 cm^−1^; ^1^H and ^13^C NMR data, see Table 1; ESIMS *m/z* 657 [M + Na]^+^; HRESIMS *m/z* 657.3258 [M + Na]^+^ (calcd for C_34_H_50_O_11_Na, 657.3245).

### Biological Activity Assays

#### Anti-TMV Assays

The half-leaf method was used to evaluate the anti-TMV activities as literatures reported [14]. Ningnanmycin, a Chinese commercial product for plant disease, obtained from Heilongjiang Qiang’er Biochemical Technology Development Company, was administered as a positive control.

#### Cytotoxicity Assay

The cytotoxicity of each compound on three cultured human cancer cell lines was tested by MTT assay. The cell lines used were SMMC-7721 (human hepatoma cells), MDA-MB-231 (triple-negative breast cancer cells), A549 (human lung cancer cell). Cell growth inhibition assay was performed as reported literatures [15]. Cisplatin was used as a positive control.
